# Canonical PKCα-immunoreactive rod bipolar cells are present in nocturnal snakes but not in diurnal snakes

**DOI:** 10.1038/s41598-026-47828-5

**Published:** 2026-06-03

**Authors:** Einat Hauzman, Juliana H. Tashiro, David J. Gower, Dora Fix Ventura, Pavel Němec, Kathleen F. Grego, Nicholas R. Casewell, Silke Haverkamp

**Affiliations:** 1 Department of Experimental Psychology, Psychology Institute, University of São Paulo, Av. Prof. Mello Moraes, 1721, São Paulo, 05508-030 Brazil; 2 Natural History Museum. Cromwell Road, London, SW7 5BD UK; 3 Department of Zoology, Faculty of Science, Charles University, Viničná 7, Prague, 128 43 Czech Republic; 4 Herpetology Laboratory, Butantan Institute, Av. Vital Brasil, 1500, São Paulo, 05503-160 Butantã Brazil; 5 Centre for Snakebite Research & Interventions, Liverpool School of Tropical Medicine, Pembroke Place. L3 5QA, Liverpool, UK; 6 Department of Computational Neuroethology, Max Planck Institute for Neurobiology of Behavior, Ludwig-Erhard-Allee 2. 53175, Bonn, Germany

**Keywords:** Bipolar cells, PKCα, Retinal circuitry, Rod pathway, Snake retina, Evolution, Neuroscience, Zoology

## Abstract

A series of studies have described aspects of outer-retinal morphology and visual opsins in different snake species. Among squamate reptiles, snakes stand out for their diversity in photoreceptor morphotypes and pronounced differences in retinal architecture between diurnal and nocturnal species. Diurnal snakes have a cone-dominated retina, lacking typical rods, but containing a transmuted, cone-like rod and exhibiting a low photoreceptor density. Nocturnal species have rod-dominated retinas with high rod densities and two or three types of cones. Despite these striking differences, no previous study has examined inner-retinal neuron types or aspects of neural circuitry in snakes. In this study, we used immunohistochemistry to investigate a specific type of bipolar cell immunoreactive to the anti-protein kinase C alpha (PKCα) antibody, a well-established marker for rod bipolar cells, and to assess its connectivity with photoreceptors in eight nocturnal and 13 diurnal species of snakes from eight families. Nocturnal snakes exhibited a canonical rod-selective PKCα-immunoreactive rod bipolar cell, whereas in diurnal species, the anti-PKCα antibody labeled a distinct population of long-wavelength-sensitive, cone-selective bipolar cells. This study reveals clear differences between diurnal and nocturnal species and highlights the remarkable plasticity and evolutionary innovation of the visual system in this group.

**Supplementary Information:**

The online version contains supplementary material available at 10.1038/s41598-026-47828-5.

## Introduction

In snakes, daily activity patterns, primarily diurnal or nocturnal, vary widely among species and are accompanied by profound changes in retinal architecture. Numerous studies have reported striking differences in the complements of photoreceptors, rods and cones, between diurnal and nocturnal snakes, even among closely related species^1–5^. Nocturnal snakes typically possess retinas with a high abundance of rods (PR0, according to the vertebrate photoreceptor nomenclature recently proposed by^6^)—highly sensitive photoreceptors adapted to scotopic (dim-light) vision—that contain the rhodopsin photopigment (RH1), alongside typically two or three types of cones, including single LWS cones (PR1), sensitive to medium or long wavelengths, and single SWS1 cones (PR4), sensitive to short wavelengths in the ultraviolet or violet range. In “basal” lineages from the paraphyletic “Henophidia” group—composed predominantly of burrowing, crepuscular or nocturnal species—only single cones are present: large LWS cones (PR1) and small SWS1 cones (PR4), whereas in lineages from the monophyletic Caenophidia, an additional double LWS cone is found, which has some differences to the double cones (PR5 and PR6) present in other tetrapods^1–5,7,8^. In contrast to the typical duplex retina pattern (with rods and cones) of nocturnal snakes, diurnal species from the Caenophidia typically have a so-called “all-cone” retina, lacking conventional rods^1–5,9–11^. In these retinas, RH1 is instead expressed in a population of transmuted (*sensu* Walls^12^, cone-like rods^5,13–15^ so that four types of cone-like photoreceptors are identified: large single LWS cones (PR1), double LWS cones (which may or may not be PR5 and PR6), small single SWS1 cones (PR4), and transmuted RH1 cone-like rods (transmuted PR0).

In the retinas of nocturnal snakes, the high density of photoreceptors and thick outer nuclear layer (comprising photoreceptor nuclei) are generally accompanied by a thinner inner nuclear layer (containing horizontal, bipolar, amacrine and glial Müller cell nuclei)^5^, suggesting a high degree of convergence from photoreceptors to bipolar cells (BCs) and elevated spatial summation—an important adaptation for scotopic vision^1^. Conversely, the retinas of diurnal species exhibit a thin outer nuclear layer (ONL) with only one or two rows of photoreceptor nuclei and a considerably thicker inner nuclear layer (INL)^5^. This arrangement reflects probable low convergence of photoreceptors to BCs and implies enhanced spatial resolution. These stark structural differences in the outer retina are suggestive of underlying divergences in the populations of inner-retinal neurons between diurnal and nocturnal snakes, as well as in their retinal circuitry. However, this has yet to be tested.

In this study, we used immunohistochemistry (IHC) to investigate a specific population of BCs immunoreactive (IR) to anti-protein kinase C alpha (PKCα) antibody, and to assess their connectivity with outer-retinal photoreceptors in diurnal and nocturnal snakes from different families. Our findings revealed distinct populations of PKCα-IR BCs in diurnal and nocturnal snakes, with a canonical PKCα-IR rod bipolar cell (RBC) present in nocturnal snakes but absent in diurnal ones. Analysis of connectivity showed that PKCα-IR BCs are rod-selective in nocturnal species, and LWS-cone-selective in diurnal species. This is the first study to explore aspects of the neural circuitry in snake retinas and provides unprecedented insights, indicating divergences in visual processing pathways between diurnal and nocturnal species—even among closely related taxa—thereby further highlighting the remarkable plasticity and evolutionary innovation of the visual system in this group of vertebrates.

## Results

### PKCα-IR bipolar cells in retinas of diurnal and nocturnal snakes

**Fig. 1:**
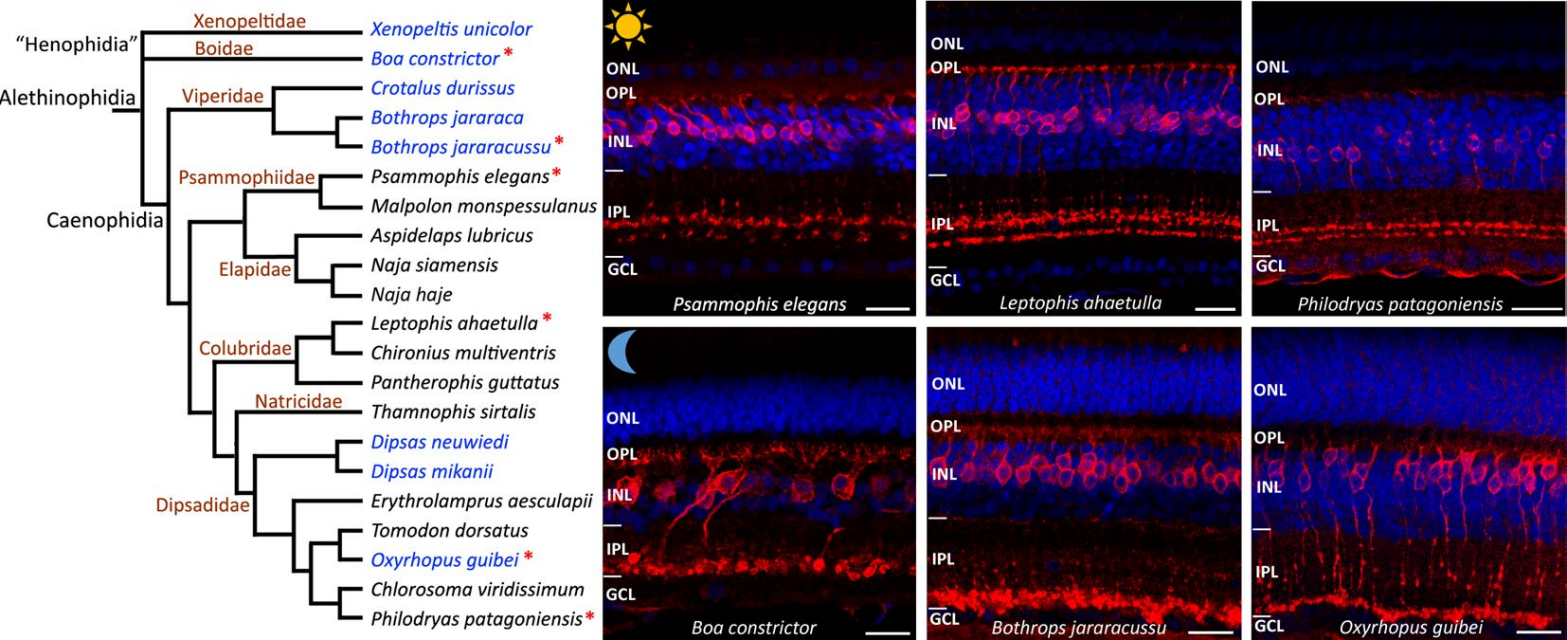
PKCα-immunoreactive bipolar cells in diurnal and nocturnal snake retinas. Phylogenetic relationships of the snake species analyzed in this study^20,21^, and retinal cross sections of three diurnal (top row) and three nocturnal (bottom row) snakes, immunolabeled with anti-PKCα antibody (red). Nocturnal species are indicated in blue text in the phylogeny. Retinal sections of the species shown in the right panel are indicated in the phylogenetic tree by red asterisks (*). In the diurnal species, the cell bodies of PKCα-IR BCs are located centrally in the inner nuclear layer (INL), and their terminals stratify in two sublaminae in the middle of the inner plexiform layer (IPL). In the nocturnal species, PKCα-IR BCs have their somata located in the outermost (distal) half of the INL, and their terminals stratify in the innermost sublamina of the IPL, close to the ganglion cell layer (GCL). Neuronal nuclei stained with DAPI, shown in blue. Differences in the thickness of the inner and outer nuclear layers (INL, ONL) are genererally observed between diurnal and nocturnal species^5^. Variation in the thickness of retinal layers seen within diurnal species and within nocturnal species depends on the retinal region. This point was not further addressed in the current study. Scale bars = 20 μm.

Immunohistochemical analyses of retinal sections revealed distinct populations of PKCα-IR BCs in diurnal and nocturnal snakes (Fig. 1; Fig. S1). In the retinas of the diurnal snakes analyzed, including 13 species from five caenophidian families, the cell bodies of PKCα-IR BCs were located in the central portion of the inner nuclear layer (INL), and their terminals stratified into two sublaminae (or three in one colubrid, *Leptophis ahaetulla*) in the center of the inner plexiform layer (IPL) (Fig. 1; Fig. S1), specifically in strata S3 and S4, considering the conventional subdivision of the IPL into five equal strata^16^, from S1 (adjacent to the INL) to S5 (at the border of the ganglion cell layer, GCL). In three diurnal species from the Elapidae family, *Aspidelaps lubricus*, *Naja haje*, and *N. siamensis*, some PKCα-IR cells had cell bodies located in the outer half of the INL, and an additional (third) band was observed in S1 (Fig. S1).

**Fig. 2:**
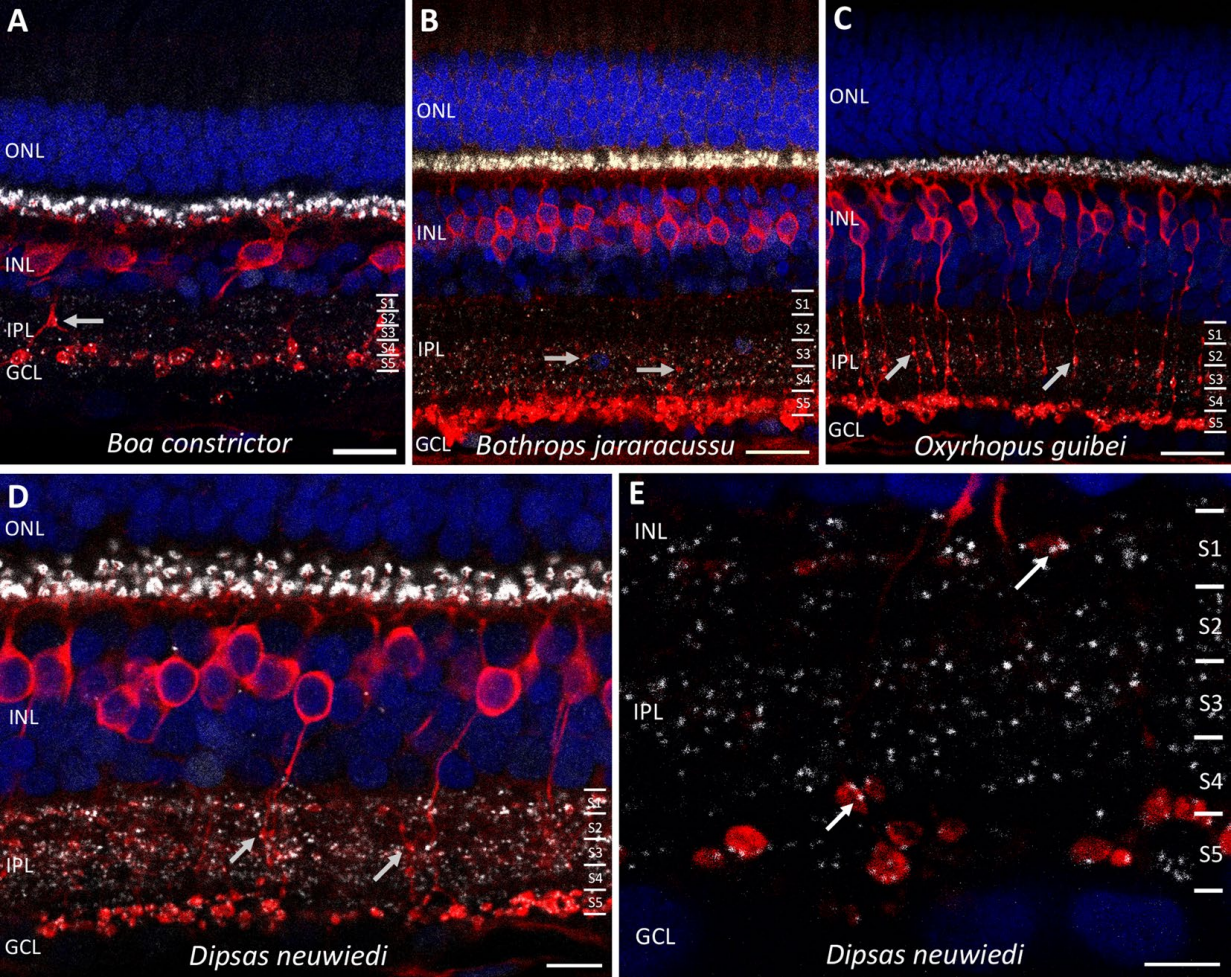
*En passant* axonal boutons in PKCα-immunoreactive bipolar cells in nocturnal snake retinas. Retinal sections of nocturnal snakes labeled with antibodies against PKCα (red) and CtBP2 (white). In all nocturnal species, PKCα-IR BCs show some boutons along their axons (grey arrows in **A**–**D**), located in different sublaminae of the inner plexiform layer (IPL), subdivided into S1 to S5. Co-staining of PKCα with CtBP2 indicates the presence of synaptic ribbons within the boutons. (**A**–**D**) Sections across the retina for four species; (**E**) Higher magnification of the IPL of *Dipsas neuwiedi*, showing the synaptic ribbons labeled by CtBP2 (arrows) within the boutons along the axons of PKCα-IR BCs. Neuronal nuclei stained with DAPI, shown in blue. Scale bars = 20 μm (**A**–**C**), 10 μm (**D**), and 5 μm (**E**).

**Fig. 3:**
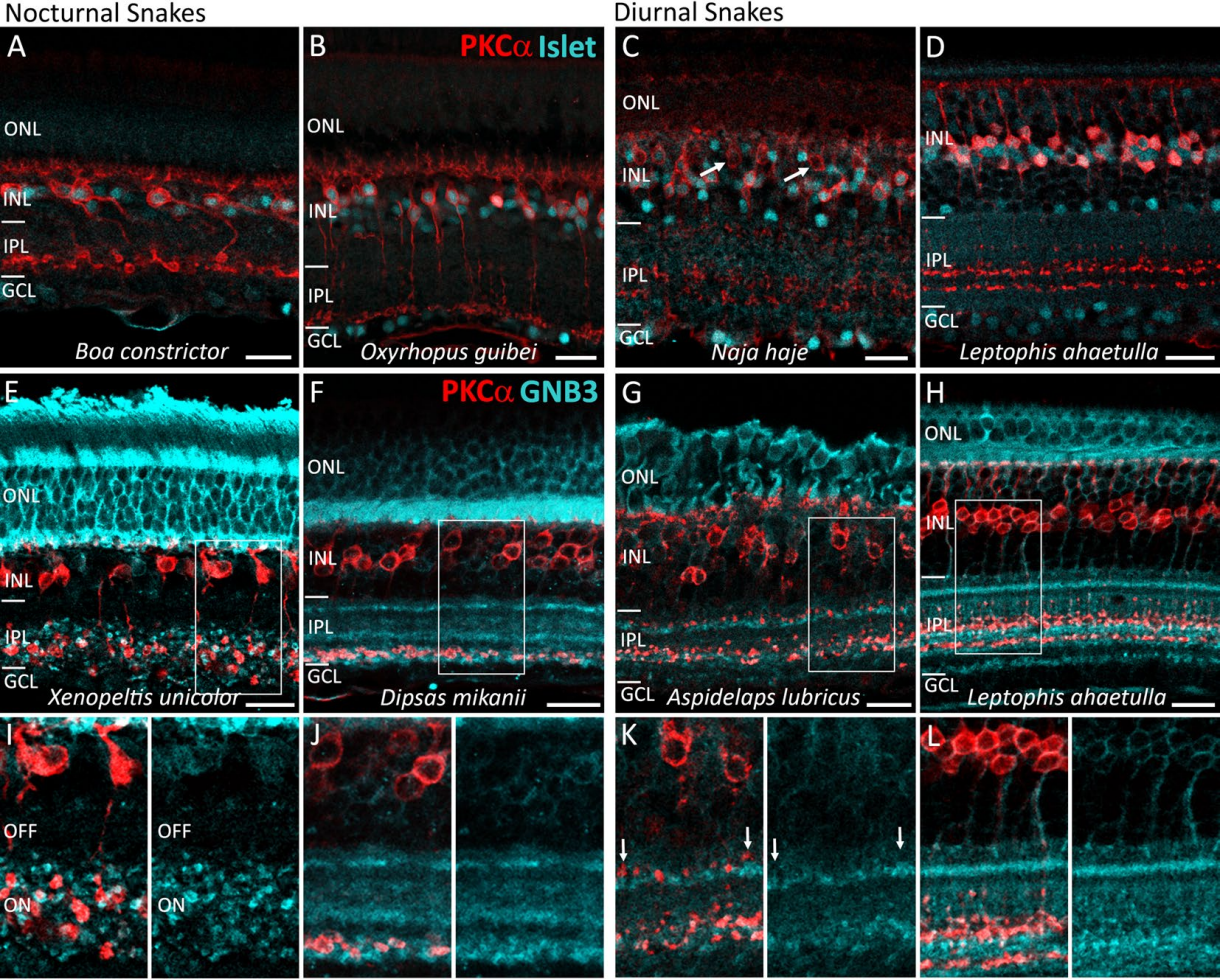
Counterstaining of PKCα-immunoreactive bipolar cells with ON bipolar cell markers in retinal sections of diurnal and nocturnal snakes. Retinal sections of nocturnal (**A**,**B**,**E**,**F**,**I**,**J**) and diurnal (**C**,**D**,**G**,**H**,**K**,**L**) snakes labeled with antibodies against PKCα (red) and Islet1 (cyan) (**A**–**D**), and against PKCα (red) and GNB3 (cyan) (**E**–**L**). In all species, PKCα-IR BCs are Islet1-positive, except in the diurnal elapid *Naja haje*, which shows a population of Islet1-negative PKCα cells (**C**: white arrows). GNB3 labeling in the inner plexiform layer (IPL) varied among nocturnal species. In the “henophidian” *Xenopeltis unicolor*, the IPL was divided into a GNB3-negative outer portion (putative OFF) and a GNB3-positive inner portion (putative ON) (**E**,**I**). In the dipsadid *Dipsas mikanii*, three narrow GNB3-positive bands were observed in S1, S3, and S5 (**F**,**J**). The PKCα terminals in S5 of nocturnal species were located in GNB3-positive bands. In the diurnal species, GNB3-positive bands were observed in sublaminae S1, S3-S4, and S5 (**G**,**H**,**K**,**L**). PKCα terminals were located in GNB3-positive bands, except in the elapid *Aspidelaps lubricus*, which showed one PKCα stratification in S1, located in a GNB3-negative band (**G**,**K**: white arrows). ONL, outer nuclear layer; INL, inner nuclear layer; GCL, ganglion cell layer. Scale bars = 20 μm.

In the nocturnal snakes analyzed, including eight species from four different families—two “henophidian” and two caenophidian families —the cell bodies of PKCα-IR BCs were located in the outer half of the INL, and their long axons extended across the entire IPL, terminating in globose terminals that stratified in the innermost sublamina (S5) of the IPL (Figs. 1, 2 and 3; Fig. S1), a typical morphology of rod bipolar cells (RBCs) described in other vertebrates^17,18^. Interestingly, in the nocturnal viperid snake *Crotalus durissus*, an additional PKCα-positive band was observed in the outermost sublamina of the IPL (S1) in the retinas of adult individuals, but not in juveniles, which showed a single stratification in S5 (Fig. S2), as observed in both adults and juveniles of the other nocturnal species analyzed.

In PKCα-IR cells of nocturnal snakes, *en passant* boutons along the axons (hereafter “boutons”) were evident from S1 to S4 (Fig. 2). Double staining with antibodies against PKCα and against CtBP2, the major protein component of the synaptic ribbons^19^, revealed the presence of these structures within the boutons, suggesting that the synaptic output of PKCα-IR cells may be multistratified (Fig. 2).

## Functional inference of the PKCα-IR BCs based on immunolabeling with ON BC markers

Bipolar cells can be functionally subdivided, according to their light responses: ON BCs respond to light with a depolarization, and OFF BCs with a hyperpolarization. To elucidate whether PKCα-IR cells are likely ON or OFF BCs, we performed double immunolabeling using antibodies against PKCα + Islet1 and PKCα + GNB3, with Islet1 and GNB3 being two known markers for ON BCs in vertebrates^22^.

In the retinas of nocturnal snake species, all PKCα-IR cells were Islet1-positive (Fig. 3A, B) and the labeling pattern for anti-GNB3 in the IPL varied among species (Fig. 3E, F, I, J). In the “henophidian” *Xenopeltis unicolor*, and in the viperid caenophidian *Bothrops jararacussu*, there was a clear division of the IPL into an outer GNB3-negative and an inner GNB3-positive half (Fig. 3E, I), and the boutons along the axons of PKCα-IR BCs were restricted to the GNB3-positive half of the IPL. In the viperid *Crotalus durissus*, the division of the IPL into an outer GNB3-negative and an inner GNB3-positive part was less distinct, but all PKCα-positive boutons along the axons, from S3 to S5, were GNB3-positive (Fig. S3). Although all PKCα-IR cells were Islet1-positive, the additional PKCα-positive band in sublamina S1 in adult individuals was GNB3-negative, suggesting that the terminals in S1 belong to an additional population of PKCα-IR cells. In the “henophidian” *Boa constrictor*, in the viperid caenophidian *Bothrops jararac*a, and in the dipsadid caenophidian *Dipsas neuwiedi*, GNB3 labeling appeared nonspecific, with strong signal throughout the entire IPL (not shown), while in the dipsadids *Dipsas mikanii* and *Oxyrhopus guibei*, three well-defined GNB3-positive bands were observed in S1, S3 and S5 (Fig. 3F, J). In all sampled nocturnal species (“henophidians” and caenophidians), the somata and the terminals of the PKCα-IR neurons were GNB3-positive. Overall, the labeling patterns indicate PKCα-IR neurons in nocturnal snakes are ON BCs.

In the retinas of diurnal snakes, double immunolabeling for PKCα and ON BC markers showed that PKCα-IR cells were Islet1-positive and their terminals stratified in GNB3-positive bands in the center of the IPL (Fig. 3C, D, G, H, K, L), indicating that they are ON BCs. GNB3-positive bands in diurnal snakes were observed in sublaminae S1, S3-S4 and S5 (Fig. 3G, H, K, L). In the three elapid caenophidians, a population of PKCα-IR cells with somata located in the outer half of the INL was not counterstained by Islet1 (for example *Naja haje*, Fig. 3C). Additionaly, the labeled stratum in S1 was GNB3-negative (*Aspidelaps lubricus*, Fig. 3G, K), indicating that elapid snakes possess an additional population of PKCα-IR OFF BCs.

## Photoreceptor terminals in nocturnal and diurnal snakes

**Fig. 4:**
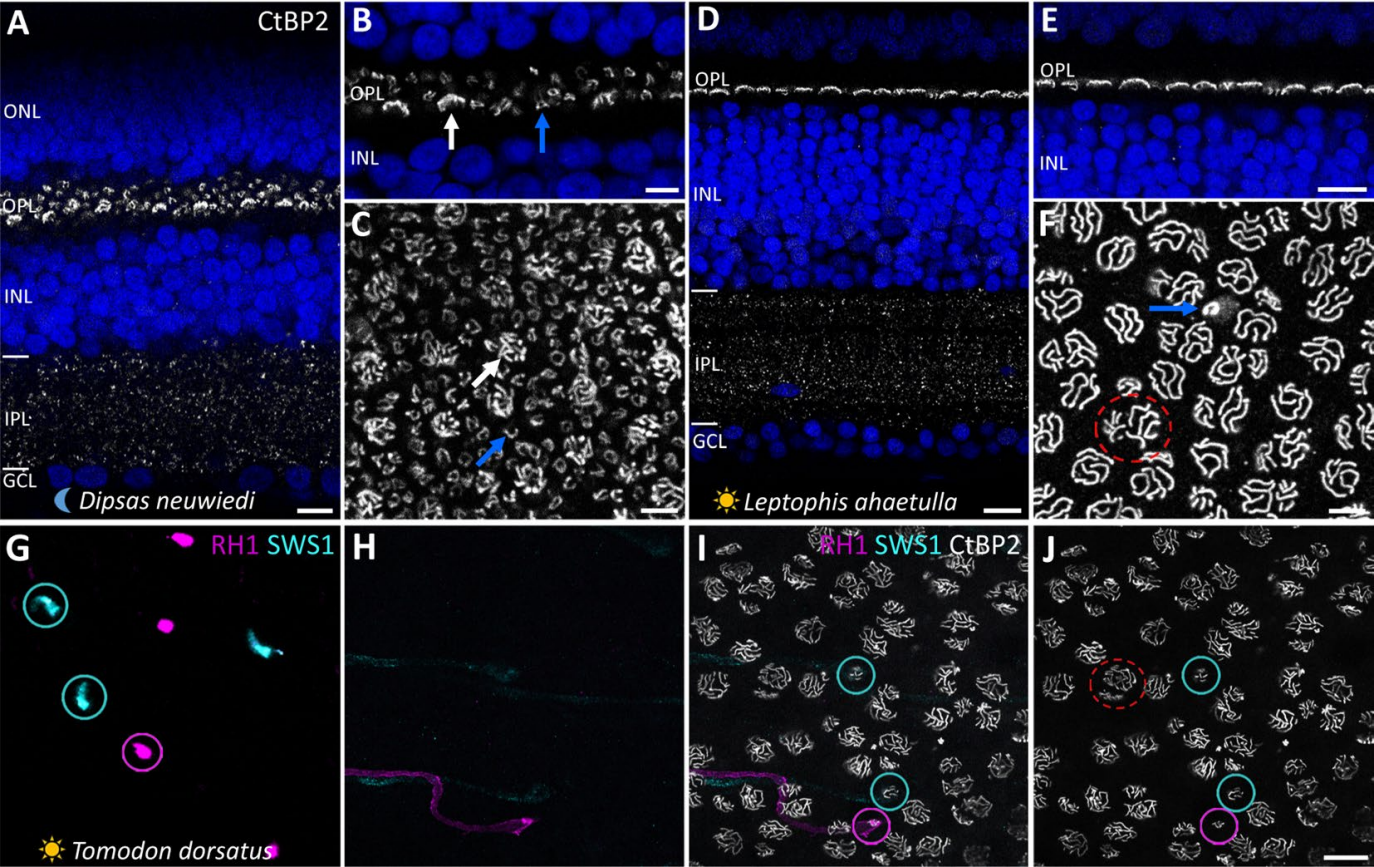
Identification of rod and cone terminals in nocturnal and diurnal snake retinas. Retinal sections (**A**,**B**,**D**,**E**) and flat-mounted retinas (**C**,**F**) of the nocturnal dipsadid *Dipsas neuwiedi* (**A**–**C**) and the diurnal colubrid *Leptophis ahaetulla* (**D**–**F**), immunolabeled with anti-CtBP2 antibody (white), which stain the synaptic ribbons in the photoreceptor terminals. Flat-mounted retinas (**G**–**J**) of the diurnal dipsadid *Tomodon dorsatus*, immunolabeled with anti-CtBP2 (white), anti-RH1 (magenta), and anti-SWS1 (cyan) antibodies. In the nocturnal species (**A**–**C**), the large cone pedicles with many synaptic ribbons (white arrows) are easily distinguishable from the small terminals (spherules) of rods, with single ribbons forming the typical horseshoe-shaped pattern (blue arrows). In the diurnal colubrid (**D**–**F**), the outer plexiform layer (OPL) has a single row of photoreceptor terminals. In the flat-mounted retina (**F**), a small terminal with a single ribbon (blue arrow), presumably from a transmuted (cone-like) rod, is distinguishable from the other, larger terminals of double cones (dashed red circle) and single cones, which contain several long ribbons. Images **G**–**J** show the same field, focused on different layers. (**G**) Outer segments of SWS1 (cyan) and RH1 (magenta) photoreceptors. The cyan and magenta circles highlight the same individual photoreceptors as shown in images **H**–**J**. (**H**) Axons of the same individual SWS1 and RH1 photoreceptors as highlighted with the circles in **G**. (**I**) Photoreceptor synaptic terminals labeled with anti-CtBP2 antibody (white). Cyan and magenta circles indicate the terminals of the same individual SWS1 and RH1 photoreceptors, respectively, as shown in images **G** and **H**. (**J**) Same image as shown in **I**, illustrating the differences in size and shape of the photoreceptor synaptic terminals: double (dashed red circle) and single LWS cones, single SWS1 cones (cyan circle), and RH1 (magenta circle) terminals. Neuronal nuclei stained with DAPI and shown in blue. ONL, outer nuclear layer; INL, inner nuclear layer; GCL, ganglion cell layer. Scale bars = 10 μm (**A**,**D**,**E**,**J**), 5 μm (**B**,**C**,**F**).

To explore the connectivity patterns of PKCα-IR BCs with the different types of photoreceptors in snake retinas, we first analyzed the photoreceptor terminals using an anti-CtBP2 antibody, in combination with anti-SWS1 and anti-RH1 antibodies, in retinal sections and flat-mounted retina pieces. In nocturnal snakes, we were able to distinguish the abundant small terminals of rods (“spherules”), which contain a single or a few (up to three) synaptic ribbons, from the rarer, large terminals of cones (“pedicles”), which each contain numerous ribbons (Fig. 4). In retinal sections of diurnal species, we observed a single row of photoreceptor terminals (Fig. 4D, E). From flat-mounted retinas, we could differentiate the synaptic terminals of four photoreceptor types using a combination of antibodies against CtBP2, SWS1, and RH1 (Fig. 4G-J). Frequently, the anti-SWS1 antibody labeled the entire photoreceptor, allowing us to pinpoint the synaptic terminals of the SWS1 cones (Fig. 4H), and occasionally, the same extensive labeling was observed with the RH1 marker, enabling identification of the four photoreceptor types based on the size and shape of their terminals (Fig. 4I): the pedicles of (1) large single LWS cones and (2) double LWS cones, easily distinguishable from each other, both displaying long synaptic ribbons (Fig. 4F); the terminals of (3) SWS1 cones, smaller, but very similar in shape to those of single LWS cones (Fig. 4J); and the terminals of (4) the photoreceptors expressing RH1 (presumably transmuted, cone-like rods), with much smaller terminals than those of the SWS1 cones, and with a single or a few ribbons (Fig. 4J).

## Connectivity patterns of photoreceptors and PKCα-IR bipolar cells

**Fig. 5:**
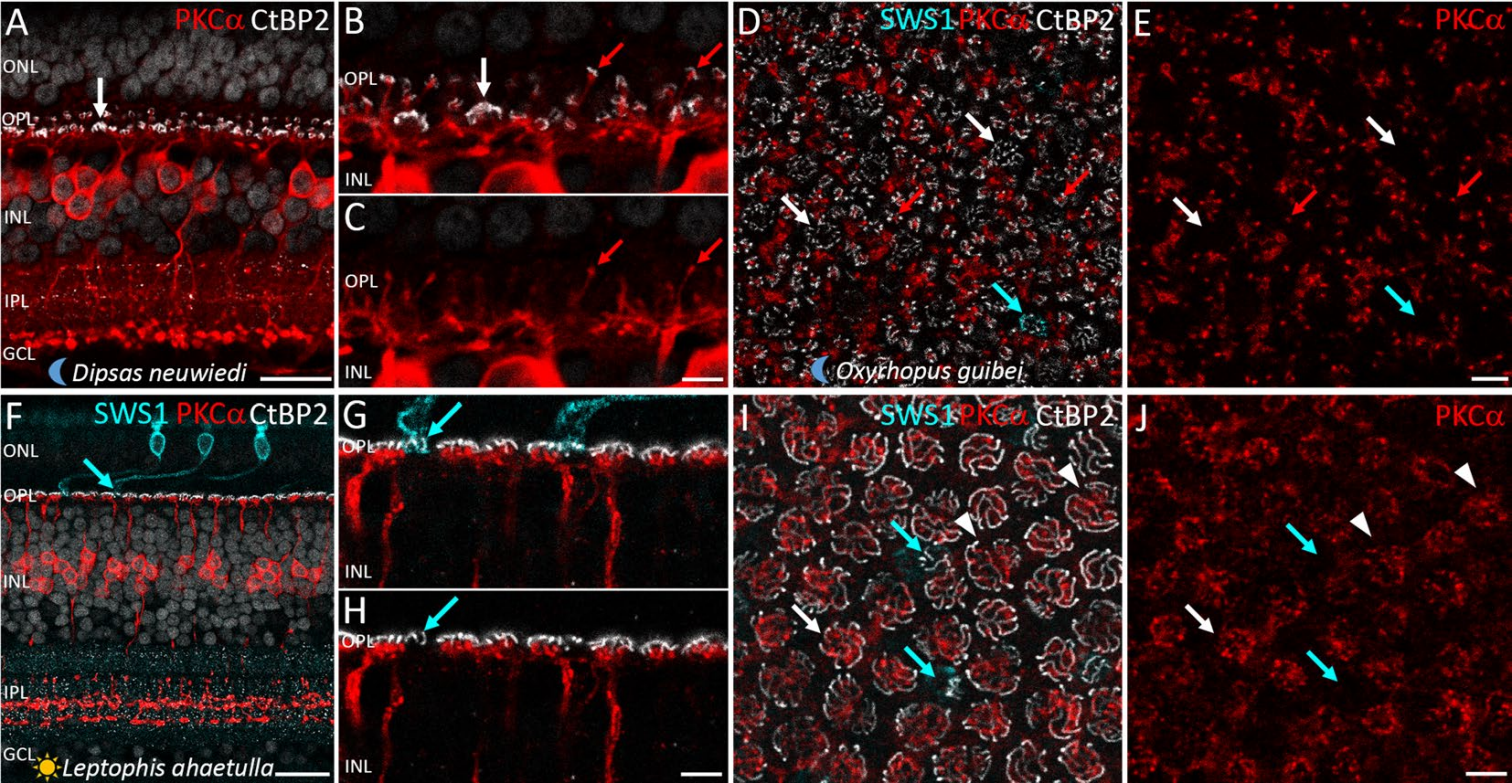
Connectivity patterns of PKCα-immunoreactive bipolar cells with photoreceptors in the retinas of nocturnal and diurnal snakes. (**A**–**E**) Retinal sections (**A**–**C**) of the nocturnal snake *Dipsas neuwiedi* immunolabeled with antibodies against PKCα (red) and CtBP2 (white), and flat-mounted retina (**D**,**E**) of the nocturnal snake *Oxyrhopus guibei*, immunolabeled with anti-PKCα (red), anti-CtBP2 (white), and anti-SWS1 (cyan). PKCα-IR BCs make synaptic contact with rods (red arrows) and avoid LWS cones (white arrows) and SWS1 cones (cyan arrows in **D**,**E**). (**F**–**J**) Retinal sections (**F**–**H**) and flat-mounted retinas (**I**,**J**) of the diurnal snake *Leptophis ahaetulla*, labeled with anti-PKCα (red), anti-CtBP2 (white), and anti-SWS1 (cyan). The PKCα-IR BCs make synaptic contact with LWS single (white arrows) and double (white arrowheads) cones, and avoid SWS1 cones (cyan arrows). Neuronal nuclei stained with DAPI and shown in grey. ONL, outer nuclear layer; OPL, outer plexiform layer; INL, inner nuclear layer; IPL, inner plexiform layer; GCL, ganglion cell layer. Scale bars = 20 μm (**A**,**F**), 5 μm (**C**,**E**,**H**,**J**).

Based on the identification of terminals of the different photoreceptor types in sections and flat-mounted retinas of diurnal and nocturnal snakes using anti-CtBP2 antibody, we were able to analyze the connectivity patterns between photoreceptors and PKCα-IR BCs. In the retinas of nocturnal “henophidian” (*X. unicolor* and *B. constrictor*) and caenophidian (*B. jararaca*, *O. guibei*, *D. mikanii* and *D. neuwiedi*) snakes analyzed, PKCα-IR BCs make selective contact with rods and avoid all cones (Fig. 5A-E). In retinas of two diurnal caenophidian snakes, the colubrid *Leptophis ahaetulla* and the psammophiid *Psammophis elegans*, we verified that PKCα-IR BCs make selective contact with single and double LWS cones and avoid small single SWS1 cones and RH1 (transmuted, cone-like) rods (Fig. 5F-J). In the other 11 diurnal species analyzed, PKCα labeling was not strong enough to visualize the dendritic tips of the BCs and their connections to the photoreceptors.

## Convergence of rods onto rod bipolar cells

Using a stereological approach for cell sampling, we analyzed the density and distribution of photoreceptors and PKCα-IR BCs, and estimated the neural convergence from rods to RBCs in the retinas of two nocturnal species from the caenophidian Viperidae family, *Bothrops jararaca* and *Crotalus durissus*. For these analyses, we used retinal wholemounts from juvenile individuals of *B. jararaca* (*n* = 4), and of juvenile (*n* = 2) and adult (*n* = 3) individuals of *C. durissus*.

The total population and mean density of cones, rods and PKCα-IR BCs of juvenile individuals of *B. jararaca*, and of juvenile and adult individuals of *C. durissus* are presented in Table 1 and Fig. S4. Based on the density values, the estimated mean convergence of rods to RBC was 13.5 ± 2.4 in juvenile *B. jararaca* retinas, with minimum and maximum convergence values across different retinal regions of approximately 6 ± 2 and 33 ± 3 rods per PKCα-IR BC, respectively (Table 1). In the retinas of juvenile *C. durissus*, the estimated mean convergence was 13 ± 1 rods per RBC. The minimum and maximum approximate convergence values across different retinal regions were 5 ± 1 and 24 ± 2 rods per RBC, respectively. In adult *C. durissus* retinas the estimated mean convergence of cells was 12.5 ± 0.7 rods per RBC. The convergence values across different retinal regions varied from approximately 6 to 31 ± 1 rods per PKCα-IR BC (Table 1).

**Fig. 6:**
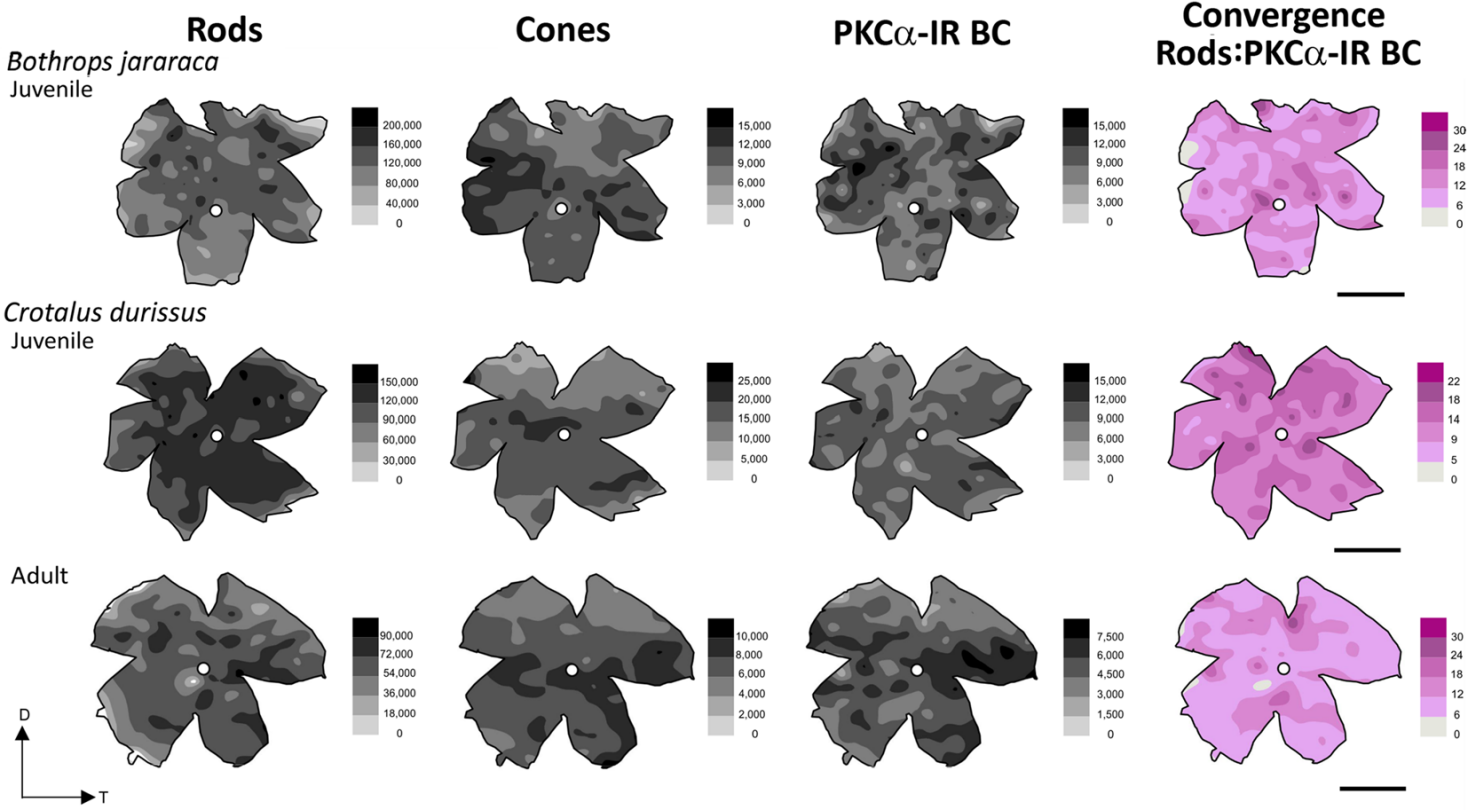
Topographic distribution of photoreceptors and PKCα-IR bipolar cells in retinas of nocturnal viperid snakes. Representative topographic maps of the retinas of *Bothrops jararaca* (juvenile) and *Crotalus durissus* (juvenile and adult), showing the distribution of rods, cones, and PKCα-IR BCs, and the estimated convergence of rods to RBC in the same retinas. Grey-shaded bars indicate the number of cells per mm². In the convergence maps (shown in magenta), the bars represent the ratio of rods to PKCα-IR BCs. The optic nerve head is depicted as a white circle. D, dorsal; T, temporal. Scale bars: 2 mm.

The isodensity maps of the retinas of *B. jararaca* showed the highest densities of cones in the nasal and ventral regions, of rods in the dorsal region, and of PKCα-IR BCs in the dorsal region, with two areas of higher cell concentration: dorso-nasal and dorso-temporal (Fig. 6). In the retinas of both adult and juvenile individuals of *C. durissus*, the isodensity maps of cones displayed a poorly defined visual streak, while rods and PKCα-IR BCs exhibited a relatively diffuse distribution, with highest concentrations in the temporal region (Fig. 6). Isodensity maps of convergence from rods to PKCα-IR BCs showed areas of higher convergence in the central and dorsal regions of the retinas in both species.

**Table 1:** Stereological assessment of the total population and mean density of rods, cones, and PKCα-IR bipolar cells in retinas of the nocturnal viperid snakes *Bothrops jararaca* and *Crotalus durissus*, and estimated convergence values from rods to PKCα-IR bipolar cells (BCs).

Species and individuals	Retinal area (mm^2^)	Total Population			Mean Density (cells mm^− 2^)			Convergence Rods: PKCα		
		Number of Rods	Number of Cones	Number of PKCα-IR BCs	Number of Rods	Number of Cones	Number of PKCα-IR BCs	Mean	Min	Max
*B. jararaca* (juveniles)										
Bjar#5-RE	20.5	3,071,310	233,801	185,675	149,820	11,405	9,057	17	8	35
Bjar#6-RE	25.4	3,153,470	220,940	199,571	124,152	8,698	7,857	13	6	34
Bjar#7-RE	23.9	2,941,231	252,031	236,049	123,064	10,545	9,877	12	4	28
Bjar#8-RE	18.9	2,679,550	263,035	229,933	141,775	13,917	12,166	12	4	36
Mean ± sd	22.2 ± 3	2,961,390 ± 207,218	242,452 ± 18,735	212,807 ± 24,117	134,703 ± 13,233	11,141 ± 2,168	9,739 ± 1,818	13.5 ± 2.4	5.5 ± 1.9	33.3 ± 3.6
*C. durissus* (juveniles)										
Cdur#5-RE	20.7	2,679,301	355,370	225,984	129,435	17,168	10,917	12	4	25
Cdur#6-RE	24.6	2,978,750	388,115	232,617	121,087	15,777	9,456	13	7	22
Mean ± sd	22.7 ± 2.8	2,829,026 ± 211,742	371,743 ± 23,154	229,301 ± 4,691	125,261 ± 5,903	16,472 ± 983	10,187 ± 1,033	12.5 ± 0.7	5.5 ± 2.1	23.5 ± 2.1
*C. durissus* (adults)										
Cdur#7-LE	66	3,162,817	329,526	255,881	47,921	4,993	3,877	13	5	31
Cdur#8-LE	49.8	3,003,416	351,218	266,120	60,225	7,053	5,336	12	5	31
Cdur#9-RE	48.6	2,776,964	367,443	231,052	57,139	7,561	4,754	14	6	30
Mean ± sd	54.8 ± 9.7	2,981,065 ± 193,895	349,395 ± 19,024	251,018 ± 18,032	55,095 ± 6,401	6,536 ± 1,359	4,656 ± 734	13 ± 1	5.3 ± 0.6	30.7 ± 0.6

sd, standard deviation.

## Discussion

We evaluated the immunoreactivity of BCs to PKCα in retinas of diurnal and nocturnal snakes from eight families and analyzed the connectivity patterns between photoreceptors and PKCα-IR BCs. In all nocturnal snakes, PKCα-IR cells exhibited a typical RBC morphology, as described for some other major vertebrate groups, including mammals^18,23^, and teleost fish^24,25^. Their somata were tightly packed in the outer half of the INL, their dendrites formed dense arborizations in the OPL, and axons extended through the entire IPL, terminating in S5. In contrast, in diurnal snakes, the PKCα-IR BCs resembled those of primarily diurnal vertebrate groups, such as birds^26^, turtles^27^, and lizards (EH, personal observation). Their somata were located in the center of the INL, and axons stratified in two or three sublaminae in the middle of the IPL.

In birds, typical RBCs have not been identified by immunohistochemistry^26^, nor through analyses of high-resolution electron microscopy volumes^28,29^. The same PKCα-IR BC pattern found here in diurnal snakes – featuring terminals stratifying in S3/S4 – has been reported across different primarily diurnal avian species^26^. Similarly, a preliminary comparative analysis of retinas from both diurnal and nocturnal lizard species revealed the same PKCα labeling pattern observed in diurnal snake (EH, personal observation), with no labeling of a typical RBC.

Among sauropsids studied to date (including snakes, lizards, turtles, alligators, and birds^30,31^), typical PKCα-IR RBCs have apparently been observed only in nocturnal snakes. Moreover, the striking difference in PKCα-IR BC types between diurnal and nocturnal snakes has not been described in other vertebrate groups. In mammals, for instance, typical PKCα-IR RBCs are present in both nocturnal^32^ and diurnal species^33^. Differences in anti-PKCα labeling patterns in mammals are instead related to the additional labeling of specific types of cone bipolar cells (CBCs) beyond the RBCs in some species^34,35,36^.

Functionally, BCs are subdivided into ON and OFF types based on their physiology and patterns of light response^37^, functional features that are associated with particular anatomical and connectivity properties. In addition, transcriptomic analyses of individual retinal cells from lamprey, zebrafish, and mouse reveal that ON and OFF cone BCs form more or less distinct molecular clusters, separated more substantially from a third cluster of (ON) RBCs^25,38,39,40^, the latter being an anatomically and molecularly specific subtype of ON BC that has been proposed to have a distinct evolutionary origin^41^.

In mammals, ON BCs depolarize in response to light and typically have axon terminals stratifying in the inner half of the IPL. In contrast, mammalian OFF BCs hyperpolarize in response to light and stratify in the outer IPL^42^. However, this varies substantially in non-mammalian vertebrates (for review see^43^). For example, OFF BCs in turtles include bistratified and tristratified types, with axons stratifying in S1 or S2 (OFF layer) and S3, S4, or S5 (ON layer)^30^.

In snakes, the diversity and functional properties of BCs remain largely unknown. Therefore, the terms ON and OFF BCs are used here only putatively, because the actual cell type requires confirmation by their responses to light stimuli. Based on the IPL functional subdivision established in mammals, we suggest that nocturnal snake PKCα-IR cells are ON BCs, given the localization of their axon terminals in S5. This interpretation is supported by immunolabeling of PKCα-IR cells with two established ON BC markers, Islet1 and GNB3^22^. All PKCα-IR BCs in nocturnal snake retinas were Islet1-positive, with axon terminals stratifying in a GNB3-positive band. Except for two nocturnal species, *Xenopeltis unicolor* and *Bothrops jararacussu*, which, like mammals, have a clear division into presumed distal OFF and proximal ON layers, GNB3 labeling in other nocturnal and diurnal snakes suggests a more complex IPL subdivision, with presumed ON sublaminae (GNB3+) intercalating presumed OFF regions and vice versa, as observed in birds^26,28^, and turtles^30^.

In nocturnal snakes, in addition to axon terminals in S5, we also observed synaptic boutons in the outer and central IPL. In mammals, multistratified RBCs are rare, with varicosities in central strata described in only a few species, including microchiropteran bats^44^, elephants^45^, and marsupials^46^. In bats, as in nocturnal snakes, presynaptic ribbons within axonal boutons indicate that RBC output occurs at multiple IPL levels^44^. In zebrafish, two types of RBCs (both ON) were distinguished, but only one has an additional axonal bouton in the middle of the IPL, suggesting that the two RBC types may contact different postsynaptic partners and participate in distinct functions, despite receiving input from the same photoreceptor types^25^.

In diurnal snakes, PKCα-IR cells were also Islet1-positive, with axon terminals located within GNB3-positive bands, indicating they are ON BCs. The only exception was in Elapidae species, which showed a population of outer-INL PKCα-IR cells that were Islet1-negative, and a PKCα-positive band in S1, which was GNB3-negative. This suggests an additional putative PKCα-IR OFF BC type in at least some elapid snakes.

In mammals, RBCs receive invaginating input primarily from rods, although some contacts with cones have been reported in some species^46,47,48^. In the few teleost fish studied to date, PKCα-IR BCs receive input from rods and all LWS cones within their dendritic field^24,25,49^. In nocturnal snakes, however, PKCα-IR BCs establish synaptic contacts exclusively with rods. Although occasional cone contacts cannot be ruled out, they are clearly neither obligatory, as in fish, nor frequent, as in some mammals.

Connectomic reconstructions from electron microscopy volumes may provide more definitive confirmation of this selective rod connectivity, as well as identify other elements of the rod pathway in snakes. In mammals, the rod pathway is well characterized: RBCs synapse onto the AII amacrine cells^50^, which forms conventional inhibitory synapses with OFF CBCs and gap junctions with ON CBCs^51^. A similar rod pathway has been described in zebrafish^25^, such that this might be conserved through much of vertebrate evolution. Whether this circuitry is conserved in snakes remains to be determined.

In diurnal colubrid and psammophiid snakes, PKCα-IR BCs make selective contacts with single and double LWS cones. The absence of contacts between these BCs and SWS1 cones suggests the presence of other BC types that selectively contact UV/violet cones, indicating the existence of specialized chromatic pathways in snakes. Lack of observation of contacts between PKCα-IR BCs and transmuted cone-like rods raises the possibility that other, non–PKCα-IR BC types may be rod-selective in diurnal snakes.

In two nocturnal viperid snakes, we estimated a mean convergence ratio of 13 rods per RBC, with maximum values up to 33:1. This high convergence suggests a strong capacity for photon capture under low-light conditions. Previously, profound differences in relative thickness of the inner and outer nuclear layers of snakes based on light microscopy were used to infer variation in photoreceptor circuitry (e.g^2,52^). A major limitation to that approach was lack of information on the identity and densities of different inner-retinal neuron types for snakes^4,]^^53^, such that the data presented here represent a first step towards that. The estimated rod-to-RBC convergences in nocturnal snakes are similar to those reported in nocturnal mammals, including cat (approximately 15:1^54^) and the marsupial *Monodelphis domestica* (26–31:1^46^). Some other mammals exhibit lower RBC densities but higher convergence ratios, such as rabbits (crepuscular) with 43–58 rods per RBC^55^ and macaques (diurnal) with ratios of 20–60:1^23^.

This study analyzed, for the first time, aspects of the inner retina of snakes using IHC in a broad comparative framework, revealing that labeling patterns of PKCα-IR BCs vary according to diel activity, regardless of phylogenetic relationships, implying substantial amounts of evolutionary convergence. In rod-dominated nocturnal snake retinas, typical PKCα-IR RBCs were observed, making selective contacts with rods, and no synaptic contacts with cones. Based on the anatomical features and connectivity patterns, we suggest that these PKCα-IR cells are orthologs of the canonical RBC of lampreys, teleost fish and mammals. In contrast, in cone-dominated diurnal snake retinas, which lack typical rods but instead possess transmuted, cone-like rods, the PKCα-IR BC displays distinct CBC-like morphology and connectivity. Based on these anatomical features, we propose that, unlike in nocturnal species, the PKCα-IR bipolar cells of diurnal snakes are not orthologs of the canonical RBC of vertebrates. This hypothesis could be tested with, for example, single-cell transcriptomic data. The occurence of different types of PKCα-IR BCs between diurnal and nocturnal species of the same major group is not common in other vertebrates and highlights the unusual and extreme adaptive plasticity of the snake retina in relation to diel activity patterns. The presence of a BC (PKCα-negative) that makes selective contact with transmuted, cone-like rods in diurnal snakes remains to be scrutinized, as does the rod downstream signaling pathway in both nocturnal and diurnal snakes. Although many aspects of snake retinal organization remain unresolved, IHC provides a powerful tool to uncover and document the diversity and connectivity of snake retinal neurons, contributing to a deeper understanding of visual processing in this group and vertebrates more generally.

## Methods

### Animals

Snakes used in this study (*n* = 36) (Table 2) were collected through fieldwork, donated by the Butantan Institute (São Paulo, Brazil), housed at the Liverpool School of Tropical Medicine (UK), or obtained commercially. The permit for specimen collection in the field was issued by the Brazilian Ministry of the Environment and the competent authority, the Chico Mendes Institute for Biodiversity Conservation (SISBIO 79155). Animals were euthanized during the daytime, using either a lethal injection of sodium thiopental (100 mg/kg) or lethal dose of ketamine and xylazine or pentobarbital sodium. All procedures were in accordance with ethical principles of animal management and experimentation established by the Brazilian Council for Control of Animal Experimentation (CONCEA), and approved by the Ethics Committee of Animal Research of the Psychology Institute, University of São Paulo, Brazil (permission number 9284040521); the Institutional Animal Care and Use Committee at Charles University in Prague (permission number UKPRF/28830/2021); or the UK Home Office and the LSTM Animal Welfare and Ethical Review Board (establishment licence X20A6D134).

We follow a classification of caenophidians in which major lineages sometimes referred to as subfamilies within Colubridae (e.g. Colubrinae) are instead considered as families (following, for example^56^,). We sampled 21 species, from two “henophidian” families and six caenophidian families: 13 diurnal species with simplex, “all-cone” retinas (in which rods, if present, are superficially cone-like), and eight nocturnal species with duplex retinas (with morphologically typical cones and rods) (Table 2). Information on specimens and voucher numbers is available in Table S1.

Following euthanasia, the eyes were enucleated, and a small radial incision was made in the dorsal region for subsequent orientation. The corneas were removed, and the eyecups were fixed for immunohistochemistry. Unless otherwise stated, eyes were collected from relatively large, presumably adult individuals. Juveniles were identified as such based on size, following information available at^57–65^.

**Table 2:** Snakes obtained for morphological analyses of PKCα immunoreactive bipolar cells. See Supporting Information (Table S1) for details of specimens.

Group	Family	Species	Daily activity	Habitat	Number of retinas used for sections	Number of retinas used for wholemounts	Number of Individuals
“Henophidia”	Xenopeltidae	*Xenopeltis unicolor*	Nocturnal	ground-dwelling	1	1	1
	Boidae	*Boa constrictor*	Nocturnal	arboreal	1	1	1
Caenophidia	Viperidae	Bothrops jararacussu*	Nocturnal	ground-dwelling	1	0	1
		Bothrops jararaca**	Nocturnal	ground-dwelling	4	4	8
		Crotalus durissus**	Nocturnal	ground-dwelling	4	5	9
	Elapidae	*Naja siamensis*	Diurnal	ground-dwelling	1	0	1
		*Naja haje*	Diurnal	ground-dwelling	1	1	1
		*Aspidelaps lubricus*	Diurnal	ground-dwelling	1	1	1
	Psammophiidae	*Malpolon monspesulanus*	Diurnal	ground-dwelling	1	0	1
		*Psammophis elegans*	Diurnal	ground-dwelling	1	1	1
	Colubridae	*Chironius multiventris*	Diurnal	arboreal	1	1	1
		*Leptophis ahaetulla*	Diurnal	arboreal	1	1	1
		*Pantherophis guttatus*	Diurnal	ground-dwelling	1	0	1
	Natricidae	*Thamnophis sirtalis*	Diurnal	ground-dwelling	1	0	1
	Dipsadidae	Chlorosoma viridissimum*	Diurnal	arboreal	1	1	1
		Oxyrhopus guibei*	Nocturnal	ground-dwelling	1	1	1
		Philodryas patagoniensis*	Diurnal	ground-dwelling	1	0	1
		*Dipsas mikanii*	Nocturnal	ground-dwelling	1	0	1
		*Dipsas neuwiedi*	Nocturnal	ground-dwelling	1	1	1
		*Tomodon dorsatus*	Diurnal	ground-dwelling	1	1	1
		*Erythrolaprus aesculapii*	Diurnal	ground-dwelling	1	0	1
Total					27	20	36

Notes. *Juvenile specimens. **Juvenile and adult specimens (see Table S1).

### Immunohistochemistry (retinal sections and wholemounts)

Eyes collected for IHC procedures were fixed in 4% paraformaldehyde (PFA) diluted in phosphate buffer saline (PBS, 10 mM, pH 7.4), for 3 h, at room temperature, and afterwards preserved in PBS with 0.05% sodium azide at 4°C. Eyes from seven species (*Xenopeltis unicolor*, *Boa constrictor*, *Naja siamensis*, *Aspidelaps lubricus*,* Psammophis elegans*, *Pantherophis guttatus*, and *Thamnophis sirtalis*) became available when animals were euthanized for another study^66^. These eyes were dissected, fixed in 4% PFA for 1 h, rinsed in PBS, incubated in a 30% sucrose solution for 24 h, and then transferred to an antifreeze solution (30% glycerol, 30% ethylene glycol, and 40% PBS). They were stored at − 20°C until further processing. One retina of each species (Table 2) was used to obtain vertical sections with a vibratome (Leica VT 1200 S) or using a cryostat (Leica CM1100). For *Crotalus durissus*, four eyes were collected for cryostat sections, two of adult individuals and two of juveniles. For vibratome sections, the retinas were embedded in 4% agarose, and sections at 70 μm thickness were collected and stored in PBS at 4°C. For cryostat sections, the retinas were emdeded in OCT mouting medium and 12 μm sections were colected onto gelatinized glass slides.

Eyes of two Viperidae species, *Bothrops jararaca* and *Crotalus durissus*, collected for wholemount preparations of the retinas and stereological assessment of cell density, were fixed in 4% PFA for variable times, ranging from 15 to 60 min (Table S2), and incubated in PBS solution containing the enzymes hyaluronidase and collagenase (Sigma-Aldrich, Cat. no. H6254 and C1764), to allow liquefaction of the vitreous humor^67^ and facilitate antibody penetration during IHC procedures. We tested different enzyme concentrations and incubation times (Table S2), depending on the consistency of the vitreous. Thereafter, the retinas were carefully dissected from the eyecups. When the pigment epithelium was too firmly attached to the retina, the pigment was bleached by incubating the retina in a 20–30% hydrogen peroxide solution diluted in PBS for 15–30 min at 55°C under 300 rpm, prior to IHC (Table S2).

For IHC, vibratome sections and whole retinas were processed free-floating. Cryostat sections were processed on the slides. The tissues were washed three times for 15 min in PBS, blocked with 10% normal donkey serum with 1% TritonX-100 in PBS for 1 h, and incubated in primary antibodies (Table 3) diluted in PBS with 1% Triton X-100. Sections were incubated overnight at room temperature and whole retinas were incubated for 3–8 nigths at 4°C (Table S2).

We identified photoreceptor terminals by using a mixture of three primary antibodies to label two out of the three visual opsins found in snakes (SWS1 and RH1), and ribeye (CtBP2), the major protein component of the synaptic ribbons^19^ (Table 3). Then, we used anti-PKCα (a well-established marker for RBCs in fish and mammals), in combination with anti-SWS1 and anti-CtBP2 antibodies on retinal sections and flat-mounted pieces of retinas, to analyze the connectivity patterns of photoreceptors and BCs. To analyze the density and distribution of PKCα-IR BCs, we incubated whole retinas of two viperid snakes (Table 2; Table S2) with anti-PKCα antibody. Finally, in retinal sections, we used combinations of antibodies against PKCα + Islet1 and against PKCα + GNB3 (Table 3), to further characterize the physiological properties of PKCα-positive BCs in snakes.

After incubation with primary antibodies (Table 3), the retinas were washed four times for 15 min each in PBS and incubated with secondary antibodies diluted in PBS with 1% Triton X-100 for 2 h, at room temperature, protected from light. Donkey secondary antibodies were conjugated with Alexa 488, Cy3, or Alexa 647 (Dianova), diluted 1:500. Retinal sections were counterstained with 4,6-diamidino-2-phenylindole (DAPI) (1:10,000; Sigma-Aldrich) to visualize the nuclear layers. Whole retinas used for density analysis of PKCα-IR BCs were incubated with a biotin–streptavidin solution to increase labeling intensity. After incubation with secondary antibodies, the retinas were washed in PBS four times for 10 min each, and incubated in 1:20 biotin solution (Jackson ImmunoResearch, Cat. no. 711-065-152), diluted in PBS with 1% Triton X-100, for 2 h, then washed in PBS four times, and incubated in 1:200 streptavidin solution (Jackson ImmunoResearch, Cat. no. 016–160-084), in PBS with 1% Triton X-100, for 2 h, and finally washed in PBS. The procedures were carried out at room temperature and protected from light. Vibratome sections and whole retinas were carefully mounted onto glass slides with Aqua-Poly/Mount (Polysciences) or Vectashield (Vector Laboratories Inc. California, United States) and a coverslip. Secondary antibody specificity was tested by omission of the primary antibodies in retinal sections. No unspecific staining was detected.

Images were obtained using a confocal laser scanning microscope (Leica TCS SP8), with the 405, 488, 554, and 647 nm lines and the PMT (photomultiplier settings). Settings were chosen to avoid cross talk between the different lines. Micrographs were acquired using an HC PL APO 40x/1.3 or HC PL APO 63×/1.4 oil immersion objective. Data were analyzed and images were adjusted for brightness and contrast using Fiji^68^.

### Antibody characterization

Table 3 lists the primary antibodies used in this study, along with their sources. For visual-opsin labeling, we used anti-SWS1 opsin and anti-rhodopsin (RH1). The specificity of the anti-SWS1 opsin antibody was tested by the manufacturer with a western blot analysis using retinal extract revealing a single band at the predicted size of ∼40 kDa. The monoclonal Ret-P1 antibody reacts with a protein of 39 kDa identified as rhodopsin.

The antibody directed against C-terminal binding protein 2 (CtBP2) was tested on retina homogenates and recognized a double band of ribeye (the retina-specific variant of CtBP2). Also, it detected an overexpressed EGFP-CtBP2 fusion construct but showed no cross-reaction with EGFP-CtBP1^69^.

PKCα is a well-established marker for RBCs in the retina of vertebrates^18^. This antibody detects an 80-kDa band on western blots of rat brain that is blocked by preincubation with the antigen but not by preincubation with corresponding peptides of other PKC isoforms; a minor band at 45 kDa is possible (manufacturer’s datasheet).

According to the manufacturer, the antibody against human Islet1 was controlled by western blot and immunocytochemistry. It recognized a single band of ∼42 kDa in lysates of human induced pluripotent stem cells differentiated into motoneurons.

The antibody against GNB3 (G-protein subunit β3) recognizes human GNB3 and GNB4. On avian tissue^26^ this antibody gave a similar staining as a rabbit GNB3 antibody described earlier^22^.

**Table 3:** Primary antibodies used in this study and their sources.

Antibody	Antigen	Host, Type	Dilution	Source; Cat#; RRID
Blue opsin (OPN1SW)	Raised against a synthetic peptide with 20 amino acids of human blue opsin (SWS1) - EFYLFKNISSVGPWDGPQYH	Goat polyclonal	1:1000	Santa Cruz Biotechnology; Cat# sc-14,363; RRID: AB_2158332
RET-P1 (RH1)	Raised against amino acids 4–10 (TEGPNFY) at the N-terminus of rat rhodopsin	Mouse monoclonal	1:1000	Millipore; Cat# MAB5316; RRID: AB_2156055
CtBP2	Synthetic peptide corresponding to aa 974 to 988 from rat Ribeye	Rabbit polyclonal	1:5000	Synaptic Systems; Cat# 193,003, RRID: AB_2086768
PKCα	Synthetic peptide corresponding to amino acids 659–672 (KVNPQFVHPILQSAV) from the C terminal variable (V5) region of rat PKCα conjugated to KLH.	Rabbit polyclonal	1:1000	Sigma-Aldrich; Cat# P4334, RRID: AB_477345
Islet1	*E. coli*-derived recombinant human Islet-1 (aa 4–349)	Goat polyclonal	1:250	R&D Systems; Cat# AF1837; RRID: AB_2126324
GNB3	Peptide sequence corresponding to aa 309–321 (SGHDNRVSCLGVT) of human transducin β chain 3	Goat polyclonal	1:200	Aviva Systems Biology; Cat# OALA06860; RRID: AB_2909439

### Stereological assessment of the density and distribution of PKCα-IR bipolar cells

The density and distribution of photoreceptors and PKCα-IR BCs were estimated from wholemounted retinas of two nocturnal species from the Viperidae family, *Bothrops jararaca* (juvenile individuals: *n* = 4), and *Crotalus durissus* (juvenile individuals: *n* = 2, adult individuals: *n* = 3) (Table 2; Table S2). To analyze cell density, we used a stereological approach based on the optical fractionator method^70^, modified for retinal wholemounts^71^. Using a motorized fluorescent microscope (DM5500B, Leica Microsystems, Germany), connected to a computer running the Stereo Investigator software (MicroBrightField, Colchester, VT; RRID: SCR_024705), we first obtained the coordinates of the retina contour with a 5x/NA 0.15 objective. Then, approximately 200 counting frames evenly spaced across the entire retinal area were positioned randomly. The sampling grid varied according to the size of the retina. Bipolar cells were counted using a 100x/1.4–0.7 oil objective, when laying entirely within the counting frame (75 × 75 μm) or when intersecting the acceptance lines (up and right), without touching the rejection lines (down and left) of the frame^72^. Photoreceptors were counted under bright light, by adjusting the focus of the microscope into the inner segments. Rods and cones were differentiated based on the size of their inner segments (Fig. S5A). Thereafter, PKCα-IR BCs were visualized by focusing on their cell bodies, under fluorescent light (Fig. S5B).

To estimate the total population of neurons (N_total_), we applied the formula ΣQ x 1/asf, where ΣQ is the sum of the total number of neurons counted, and asf, is the area of sampling fraction, which corresponds to the ratio between the counting frame and the sampling grid^70^. The stereological parameters used to estimate the number of cells of each retina are described in Table S3. For each cell type counted, we calculated the Scheaffer coefficient of error (CE) and considered an acceptable value of < 0.10^73^.

## Supplementary Information

Below is the link to the electronic supplementary material.

Supplementary Material 1

## Data Availability

The data that support the findings of this study are included in the manuscript, in supplementary material and are available from the corresponding author upon reasonable request.
