# Supplementary material for: Canonical PKCα-immunoreactive rod bipolar cells are present in nocturnal snakes but not in diurnal snakes
Source: Sci Rep. 2026 Jun 3;16:17176. doi: 10.1038/s41598-026-47828-5 (PMC13233981; doi:10.1038/s41598-026-47828-5)
Supplement: Supplementary file 1 — Supplementary Material 1 [file 41598_2026_47828_MOESM1_ESM.pdf]

Canonical PKCα-immunoreactive rod bipolar cells are present in nocturnal snakes but not in diurnal snakes.

Einat Hauzman, Juliana H. Tashiro, David J. Gower, Dora Fix Ventura, Pavel Němec, Kathleen F. Grego, Nicholas R. Casewell, Silke Haverkamp

Supplementary Information

Table S1. Snake specimens collected and voucher numbers.

Table S2. Tissue preparation protocols prior to immunohistochemistry (IHC), in whole retinas of the nocturnal viperid snakes *Bothrops jararaca* and *Crotalus durissus*.

Table S3. Stereological parameters used to estimate the number and distribution of photoreceptors and PKCα-IR bipolar cells in retinas of the viperid snakes *Bothrops jararaca* and *Crotalus durissus*.

Fig. S1. Retinal cross-sections of nocturnal and diurnal snakes, immunolabeled with anti-PKCα antibody.

Fig. S2. Retinal sections of adult and juvenile individuals of *Crotalus durissus* labeled with anti-PKCα antibody.

Fig. S3. Retinal sections of adult and juvenile individuals of *Crotalus durissus* labeled with antibodies anti-PKCα + anti-GNB3, and with antibodies anti-PKCα + anti-Islet.

Fig. S4. Mean densities (cell mm^{-2}) and total population of rods, cones and PKCα-IR bipolar cells in retinas of juvenile individuals of *Bothrops jararaca* (●), and juvenile (■) and adult (♦) individuals of *Crotalus durissus*.

Fig. S5. Sampling field of a wholemounted retina of *Bothrops jararaca*.

Table S1. Snake specimens collected and voucher numbers. Vernacular names for species from Reptile Database (Uetz et al. 2025), or proposed by Hauzman et al. (2025) (species indicated with two asterisks, **). Juvenile specimens indicated with one asterisk (*). Nocturnal taxa shaded grey.

Group	Family	Species	Vernacular name	ID	Obtained from	Native to	Sex	Size	Deposit/Voucher
“Henophidia”	Xenopeltidae	*Xenopeltis unicolor*	Sunbeam Snake	XUN1	Prague, Czechia	Asia	F	SVL+TL=715+73 mm; m= 178 g	DZCHU-XUN1
	Boidae	*Boa constrictor*	Boa Constrictor	BCON1	Prague, Czechia	South America	M	SVL+TL=2,190+310 mm; m=11,90 g	DZCHU-BCON1
Caenophidia	Viperidae	*Bothrops jararaca*	Jararaca, Lancehead	Bjar#3_230705	Itu, São Paulo, Brazil	South America	M	SVL+TL= 665+110 mm	-
				Bjar#1_220221.3*	São Paulo, Brazil		-	-	-
				Bjar#4_230511*	Ibiuna, São Paulo, Brazil		M	SVL+TL= 440+78 mm	-
				Bjar#5_230131.18*	São Paulo, Brazil		M	SVL+TL= 270+50 mm; m= 9.7g	-
				Bjar#6_230131.19*	São Paulo, Brazil		F	SVL+TL= 275+55 mm; m= 10.7g	-
				Bjar#7_230131.24*	São Paulo, Brazil		F	SVL+TL= 260+50 mm; m= 9.6g	-
				Bjar#8_230131.28*	São Paulo, Brazil		M	SVL+TL= 260+45 mm; m=9.3g	-
				Bjar_230112.1*	São Paulo, Brazil		F	SVL+TL=425+48 mm; m=22.3g	-
		Bothrops jararacussu	Jararacussu	Bjus_230112.1*	São Paulo, Brazil	South America	M	SVL+TL=303+35.5 mm; m=16.4g	-
		Crotalus durissus	Central American Rattlesnake	Cdur#3_230705.35	Itu, São Paulo, Brazil	South America	F	SVL+TL= 930+70 mm	-
				Cdur#4_230705.21	Itu, São Paulo, Brazil		F	SVL+TL= 670+60 mm	-
				Cdur#7_230705.23	Itu, São Paulo, Brazil		F	SVL+TL= 1000+70 mm	-
				Cdur#8_230705.24	Itu, São Paulo, Brazil		M	SVL+TL= 970+90 mm	-
				Cdur#9_230705.27	Itu, São Paulo, Brazil		M	SVL+TL= 950+120 mm	-
				Cdur#1_220221*	São Paulo, Brazil		-	-	-
				Cdur#2_230118.1*	São Paulo, Brazil		F	SVL+TL= 342+36.5mm	-
				Cdur#5_230112.5*	São Paulo, Brazil		M	SVL+TL= 295+32.5 mm; m=19.5g	-
				Cdur#6_220131.13*	São Paulo, Brazil		F	SVL+TL= 315+35 mm; m=36.1g	-
	Elapidae	*Naja siamensis*	Indo-Chinese Spitting Cobra	NSI1	Prague, Czechia	Asia	M	SVL+TL=1298+225 mm; m=1083 g	-
		Naja haje	Egyptian Cobra	NivZAF004	Liverpool, UK	Africa		-	-
		Aspidelaps lubricus	Angolan Coral Snake	ALU1	Prague, Czechia	Africa	F	SVL+TL=555+43 mm; m=136 g	DZCHU-ALU1
	Psammophiidae	*Malpolon monspesulanus*	Montpellier Snake	Malp1		Africa/Europe		-	NHMUK 2022.07576
		Psammophis elegans	Elegant Sand Racer	PEL1	Prague,Czechia	Africa	M	SVL+TL=1000+600 mm; m=119g	DZCHU-PEL1
	Colubridae	*Chironius multiventris*	South American Sipo	Cmulti220121_LE	Manaus, Brazil	South America			UFAM – CZPB-RP-1075
		Leptophis ahaetulla	(Giant) Parrot Snake	Lahe220118.1	Manaus, Brazil	South America	M	SVL+TL=635+41 mm	UFAM – CZPB-RP-1072
		Pantherophis guttatus	Red Cornsnake	EGU1	Prague, Czechia	North America	M	SVL+TL=1208+208 mm; m=584g	DZCHU-EGU1
	Natricidae	*Thamnophis sirtalis*	Common Garter Snake	TSI1	Prague, Czechia	North America	F	SVL+TL=543+172 mm; m=50g	DZCHU-TSI1
	Dipsadidae	*Chlorosoma viridissimum*	Common Green Racer	Cvir220117*	Manaus, Brazil	South America	F	SVL+TL=342+120 mm	UFAM – CZPB-RP-1071
		Oxyrhopus guibei	Guibé's False Coral Snake**	Oguib221216*	São Paulo, Brazil	South America		SVL+TL=200+50 mm; m=5.2 g	IBSP95379
		Philodryas patagoniensis	Patagonian Green racer	Ppat230203*	São Paulo, Brazil	South America	M	SVL+TL=350+140 mm; m=14 g	IBSP95378
		Dipsas mikanii	Mikan's Tree Snake**	Smik221216	São Paulo, Brazil	South America	F	SVL+TL=430+75 mm; m=26.7g	IBSP95376
		Dipsas neuwiedi	Neuwied's Tree Snake	Sneu221216	São Paulo, Brazil	South America	F	SVL+TL=480+125 mm	IBSP95377

		Tomodon dorsatus	Pampas Snake	Tdor211125	São Paulo, Brazil	South America		SVL+TL=600+200 mm; m=67.4 g	IBSP95375
		Erythrolaprus aesculapii	Aesculapian False Coral Snake	Eaesc311023	Araçariguama, São Paulo, Brazil	South America	-	SVL+TL= 474+65 mm; 23.45g	-

Notes. SVL, snout-vent length; TL, tail length; m, mass; DZCHU, Department of Zoology, Charles University, Prague; NHMUK, Natural History Museum, London; UFAM, Universidade Federal do Amazonas; IBSP, Instituto Butantan São Paulo.

Table S2. Tissue preparation protocols prior to immunohistochemistry (IHC), in whole retinas of the nocturnal viperid snakes *Bothrops jararaca* and *Crotalus durissus*, used for stereological analysis of cell density.

Species/ Individuals	Fixation time (4% PFA)	Hyaluronidase (H)/ Collagenase (C)	Bleaching	Incubation in anti-PKCα antibody
B. jararaca (juveniles)				
Bjar#5-RE	1h	0,1mg/ml H – 4 days, 37°C	-	4 days
Bjar#6-RE	1h	0,1mg/ml H – 2 days, 37°C	-	4 days
Bjar#7-RE	1h	0,1mg/ml H – 2 days, 37°C	-	4 days
Bjar#8-RE	1h	0,5mg/ml H – 4 days, 37°C	-	8 days
C. durissus (juveniles)				
Cdur#5-RE	30 min	-	20% HPS + 0,5mg/ml H+C, 30 min, 55°C, 300 rpm	5 days
Cdur#6-RE	40 min	0,5mg/ml C + 0,5mg/ml H – 4 days, 37°C	20% HPS + 0,5mg/ml H+C, 15 min, 55°C, 300 rpm	4 days
C. durissus (adults)				
Cdur#7-LE	15 min	-	30% HPS, 55°C, 300 rpm	3 days
Cdur#8-LE	15 min	0,5mg/ml C + 0,5mg/ml H – 1 day, 25°C	30% HPS, 55°C, 300 rpm	3 days
Cdur#9-RE	30 min*	0,5mg/ml C + 0,5mg/ml H – 30 min, 25°C	-	5 days

HPS, hydrogen peroxide solution. *Incubation with collagenase 0,5mg/ml for 20 min before fixation.

Table S3. Stereological parameters used to estimate the number and distribution of photoreceptors and PKCα-IR bipolar cells in retinas of the viperid snakes *Bothrops jararaca* and *Crotalus durissus*, using the optical fractionator method.

Species/ Retina (ID)	Retinal Area (mm^2)	Counting Frame (µm x µm)	Grid (µm x µm)	Area of sampling fraction (asf)	Number of sites counted
B. jararaca (juveniles)					
Bjar#5-RE	20.5	75 x 75	300x300	0.0573	209
Bjar#6-RE	25.4	75 x 75	300x300	0.0372	168
Bjar#7-RE	23.9	75 x 75	300x300	0.0572	243
Bjar#8-RE	18.9	75 x 75	300x300	0.0530	178
C. durissus (juveniles)					
Cdur#5-RE	20.7	75 x 75	300x300	0.0565	208
Cdur#6-RE	24.6	75 x 75	350x350	0.0409	179
C. durissus (adults)					
Cdur#7-LE	66	75 x 75	550x550	0.0169	198
Cdur#8-LE	49.8	75 x 75	500x500	0.0204	181
Cdur#9-RE	48.6	75 x 75	500x500	0.0184	159

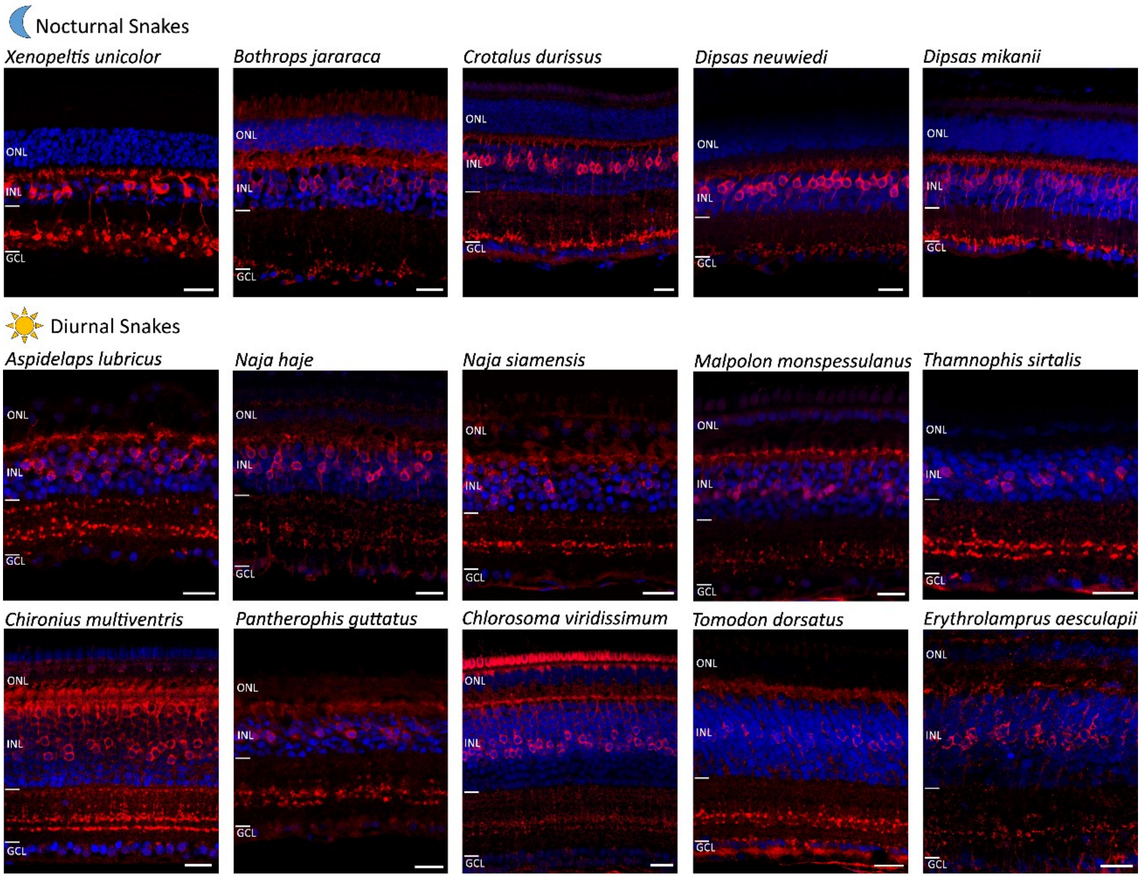

Fig. S1. Retinal cross-sections of nocturnal and diurnal snakes, immunolabeled with anti-PKCα antibody (red). In the nocturnal species, PKCα-IR bipolar cells have their somata located in the outermost (distal) half of the inner nuclear layer (INL), and their terminals stratify in the innermost sublamina of the inner plexiform layer (IPL), close to the ganglion cell layer (GCL). In the diurnal species, the cell bodies of PKCα-IR bipolar cells are located centrally in the INL, and their terminals stratify in two sublaminae in the middle of the IPL. In the elapid snakes *Aspidelaps lubricus*, *Naja haje* and *Naja siamensis*, some cell bodies are located in the outer half of the INL and an additional band is observed in the outermost sublamina of the IPL, close to the INL. Some PKCα labeling was also observed in the ganglion cell layer (GCL) of some diurnal species, as in *Chironius multiventris* and *Tomodon dorsatus*. Double staining with antibodies against PKCα and against CtBP2, the major protein component of the synaptic ribbons, shows absence of co-staining in the GCL, indicating that this PKCα labeling in the GCL is likely unspecific (data not shown). The outer and inner borders of the IPL are indicated by horizontal bars in each image. Neuronal nuclei stained with DAPI, shown in blue. ONL, outer nuclear layer; GCL, ganglion cell layer. Scale bars = 20 µm.

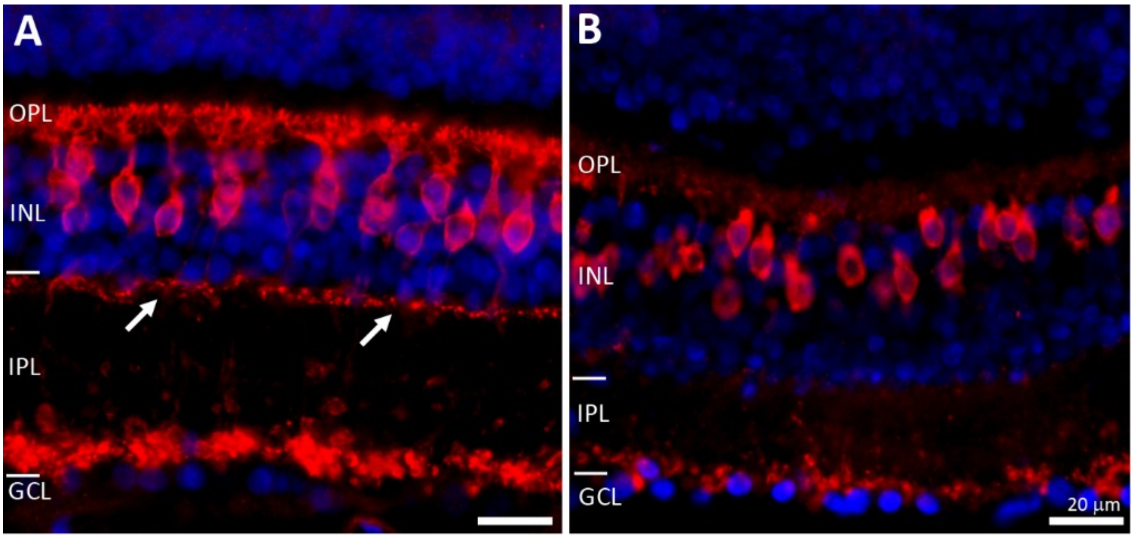

Fig. S2. Retinal sections of adult (A) and juvenile (B) individuals of *Crotalus durissus* labeled with anti-PKCα antibody (red). In the adult (A) the PKCα-IR bipolar cells stratify in two sublayers of the inner plexiform layer (IPL), S1 (arrows) and S5, close to the ganglion cell layer (GCL). In the juvenile (B), PKCα-IR bipolar cells stratify only in S5. Neuronal nuclei stained with DAPI (blue). OPL, outer plexiform layer; INL, inner nuclear layer.

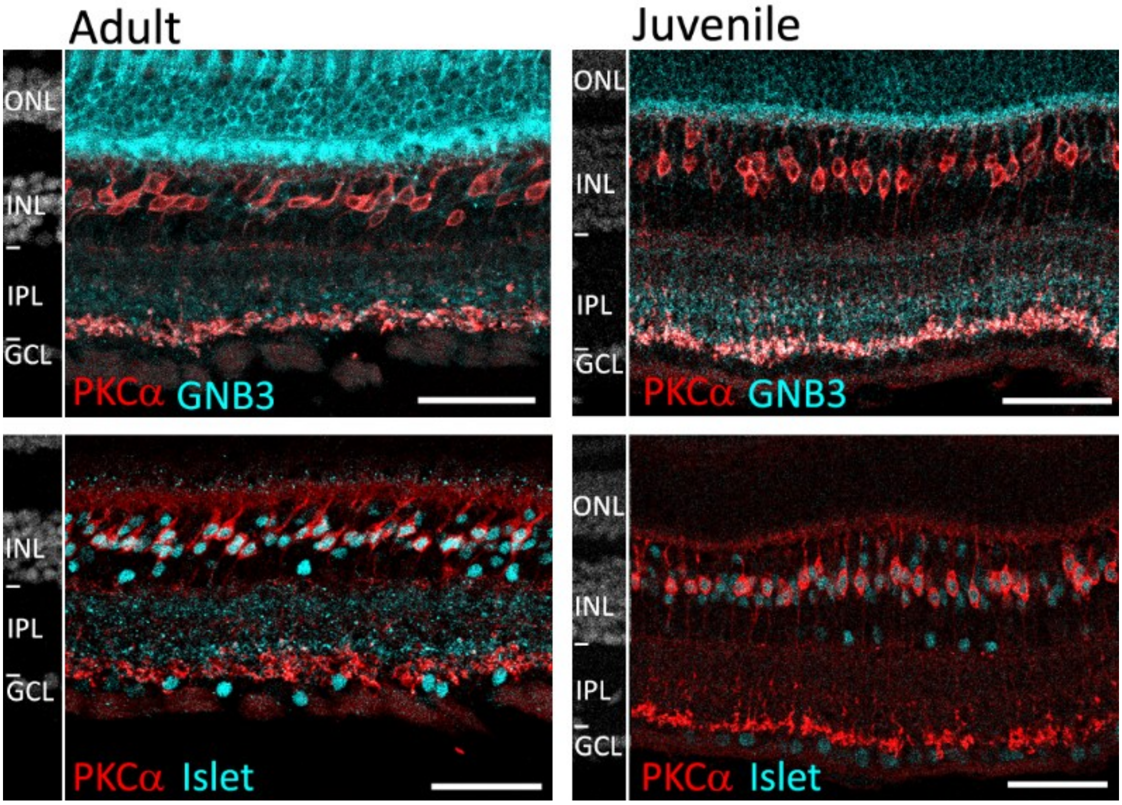

Fig. S3. Retinal sections of adult (left) and juvenile (right) individuals of the nocturnal viperid snake *Crotalus durissus* labeled with antibodies anti-PKCα (red) + anti-GNB3 (cyan) (upper images), and with antibodies anti-PKCα (red) + anti-Islet (cyan) (lower images). Neuronal nuclei stained with DAPI (gray). ONL, outer nuclear layer; INL, inner nuclear layer; IPL, inner plexiform layer; GCL, ganglion cell layer. Scale bars = 50 µm.

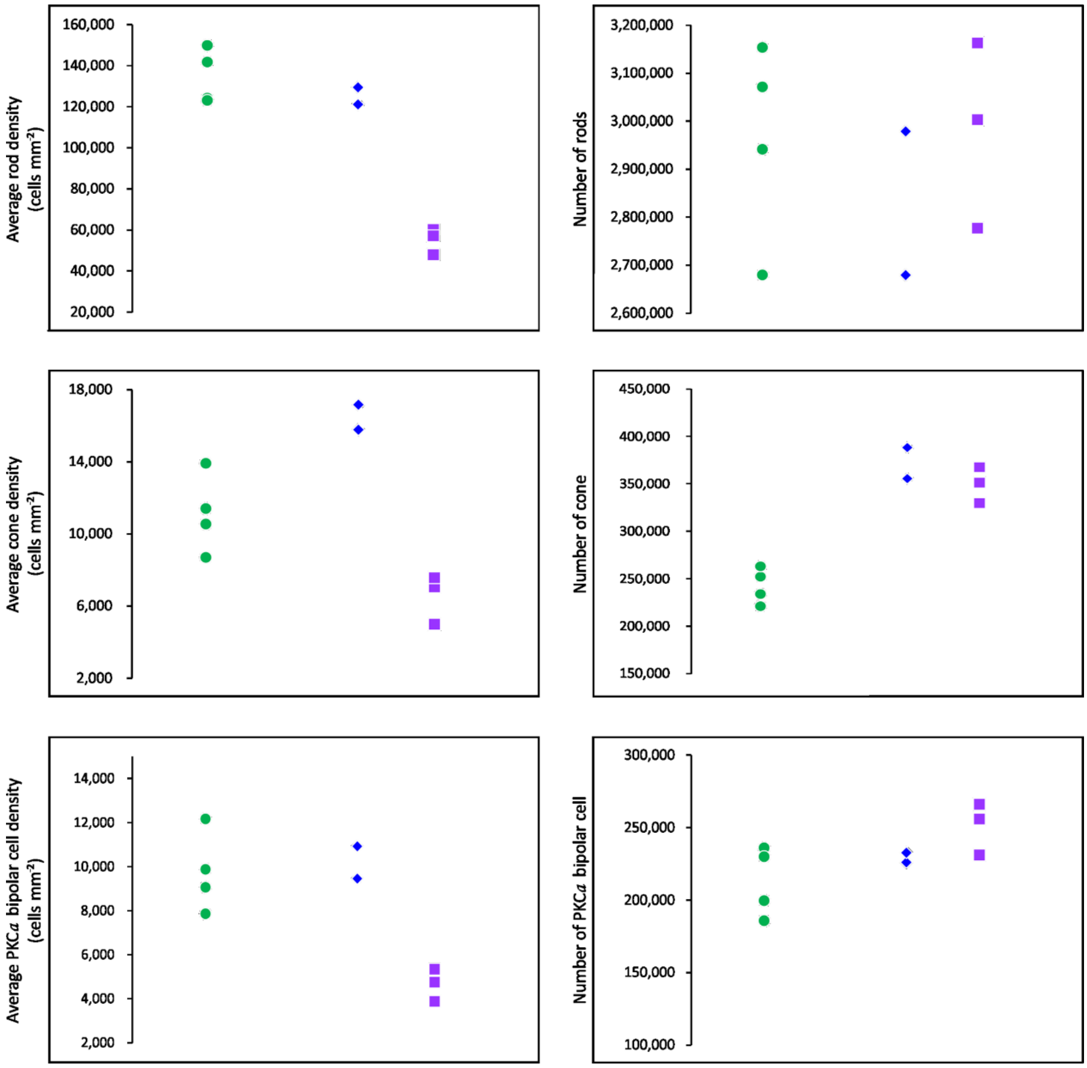

Fig. S4. Mean densities (cell mm^{-2}) and total population of rods, cones and PKCα-IR bipolar cells in retinas of juvenile individuals of *Bothrops jararaca* (●), and juvenile (■) and adult (♦) individuals of *Crotalus durissus*.

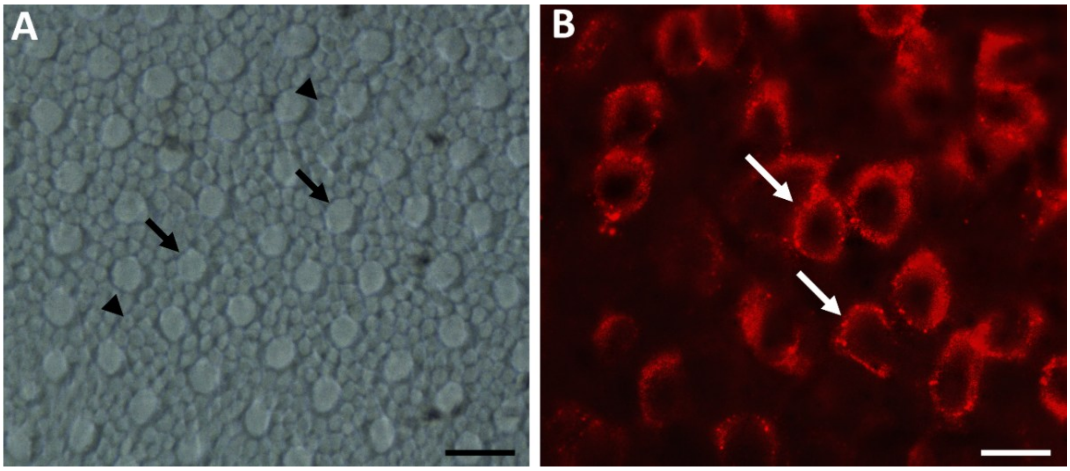

Fig. S5. Sampling field of a wholemounted retina of *Bothrops jararaca*. (**A**) Photoreceptors mosaic showing the large inner segments of cones (black arrows) and the small inner segments of rods (black arrowheads). (**B**) Somata of PKCα-IR bipolar cells (white arrows). Scale bars 10 μm.
